# The fast–slow continuum of longevity among yellow-bellied toad populations (*Bombina variegata*): intrinsic and extrinsic drivers of variation

**DOI:** 10.7717/peerj.8233

**Published:** 2019-12-16

**Authors:** Alena Marcella Hantzschmann, Birgit Gollmann, Günter Gollmann, Ulrich Sinsch

**Affiliations:** 1 Department of Biology, University of Koblenz-Landau, Koblenz, Germany; 2 Department of Limnology and Bio-Oceanography, University of Vienna, Wien, Austria; 3 Department of Theoretical Biology, University of Vienna, Wien, Austria

**Keywords:** Fast-slow continuum, Life history, Demography, Longevity, Adult mortality, Skeletochronology, Climate, Growth pattern, Palatability

## Abstract

Yellow-bellied toad populations (*Bombina variegata*) show a wide fast–slow continuum of the life-history trait longevity ranging from 5 to 23 years. We investigated populations in Germany (*n* = 8) and Austria (*n* = 1) to determine their position within the continuum of longevity and the potential drivers of adult survival at the local and the continental scale. Intrinsic and extrinsic factors considered were local weather, nutritional state, allocation of ingested energy to somatic growth, pathogen prevalence, and geographical clines (latitude, altitude, and longitude). Capture-mark-recapture (CMR) monitoring and direct age assessment by skeletochronology allowed for reliable estimates of longevity and adult survival. Raw and corrected recapture rates as well as a probabilistic estimate of the lifespan of the eldest 1% adults of a cohort (CMR data) were used as surrogates for adult survival and thus longevity in a population. Additionally, survival rates were calculated from static life tables based on the age structure (skeletochronological data) of eight populations. Populations in Germany were short-lived with a maximum lifespan of annual cohorts varying from 5 to 8 years, whereas the population in Austria was long-lived with a cohort longevity of 13 to 23 years. We provide evidence that annual survival rates and longevity differ among years and between short- and long-lived populations, but there was no decrease of survival in older toads (i.e. absence of senescence). Variation of weather among years accounted for 90.7% of variance in annual survival rates of short-lived populations, whereas the sources of variation in the long-lived population remained unidentified. At the continental scale, longevity variation among *B. variegata* populations studied so far did not correspond to geographical clines or climate variation. Therefore, we propose that a population’s position within the fast–slow continuum integrates the response to local environmental stochasticity (extrinsic source of variation) and the efficiency of chemical antipredator protection determining the magnitude of longevity (intrinsic source of variation).

## Introduction

Variation of life-history traits related to reproduction is shaped by the interaction of environmental variability and constraints of the organism such as trade-offs between size, fecundity and longevity (Stearns, 2000). Most of life-history variation among species falls on a fast–slow continuum, with low fecundity, slow growth and long lifespan at one end and the opposite combination of traits at the other end (Stearns, 1983; Gaillard et al., 1989; Ricklefs & Wikelski, 2002). In mammals, there are strong size-independent correlations among life-history variables, e.g., species with late age at maturity have often low rates of juvenile and adult mortality and small litters with large neonates (Bielby et al., 2007; for an exception see Kraus et al., 2005). The key driver of variation in life-history traits seems to be mortality (Read & Harvey, 1989; Promislow & Harvey, 1990; Cayuela et al., 2015). Evolutionary theories of ageing postulate that mortality rates determine the range of longevity, a trait shaped by selection on genetic processes, behaviour and physiology (Kirkwood & Austad, 2000; Wilkinson & South, 2002; Bonsall & Mangel, 2004). High levels of adult mortality drive species to a fast lifestyle with early sexual maturation and short lifespan, whereas low extrinsic mortality favours longevity (Reznick & Endler, 1982; Sæther, Ringsby & Røskaft, 1996; Kirkwood & Austad, 2000; Kraus et al., 2005). Long-lived species are commonly less affected by climate variability than their short-lived counterparts because longevity buffers adult survival against temporal environmental variation (Gaillard & Yoccoz, 2003; Morris et al., 2008).

The fast–slow life-history continuum of amphibian species results in a broad range of maximum lifespans, with larger, nocturnal, and poisonous species tending to live longer (Stark & Meiri, 2018). In contrast to the trends in birds and mammals, the main driver of longevity, annual adult mortality, is sometimes highly variable among conspecific populations (Stearns, 1983; Stearns, 2000; Berven & Gill, 1983; Sinsch et al., 2010; Sinsch, Pelster & Ludwig, 2015; Cayuela et al., 2017), and exceptionally within a population (Becker et al., 2018). Adult survival, and consequently the position of a population within the fast–slow longevity continuum is affected by the complex interactions of extrinsic and intrinsic factors, e.g., weather and climate, food availability and allocation of ingested energy, predation and chemical defences, and pathogens. Environmental stochasticity mediated by weather variability and local climate is one of the most influential extrinsic factors for survival due to water permeability of amphibian skin and ectothermic metabolism (Hillman et al., 2008; Tuljapurkar, Gaillard & Coulson, 2009; Hjernquist et al., 2012; Cayuela et al., 2016; Cayuela et al., 2017; Becker et al., 2018). Climate also shapes habitat predictability, i.e., presence and location of breeding and shelter sites (Piha et al., 2007; Cayuela et al., 2019a), and food availability, i.e., energy resources for somatic growth and reproduction (Reading, 2007; Mills et al., 2008). Predation contributes considerably to mortality in amphibians (Lima & Dill, 1990; Grant, Ransom & Liebgold, 2018), but unpalatability determined by chemical defences (skin toxins) increases survival (Darst, Cummings & Cannatella, 2006; Hettyey et al., 2019). Pathogens, i.e., chytrid fungi, Ranavirus, and parasites, may reduce viability of hosts or cause their death (Spitzen-van der Sluijs et al., 2017; Campbell et al., 2018; Becker et al., 2019; Sinsch et al., 2019). Consequently, disentangling the multifactorial sources of variation in annual survival and longevity requires determining the specific influence of as many involved factors as possible.

Wide among-population variation of adult survival rates and longevity makes the yellow-bellied toad *Bombina variegata* an excellent model organism to analyse an intraspecific fast–slow continuum of demographic life-history traits (Gollmann & Gollmann, 2012). The maximum lifespan of this toad is up to 23 years in the field (Di Cerbo et al., 2011; this study) and 27–29 years in captivity, i.e., in the absence of extrinsic mortality factors (Mertens, 1964; Mertens, 1970; Abbühl & Durrer, 1998). In Amphibia, it is rare that longevity of free-ranging individuals exceeds 20 years (verified exceptions: *Xenopus laevis*: 20 years; Measey & Tinsley, 1998; *Salamandra salamandra*: 20–25 years; Feldmann, 1987; Rebelo & Leclair, 2003; Heine & Thiesmeier, 2010; *Calotriton asper*: 26 years; Montori, 1990; *Taricha rivularis*: 20-30 years; Hedgecock, 1978). Chemical defense based on bioactive substances (e.g., alkaloids, peptides in *Bombina* spp.) in the skin seems to promote longevity (Kiss & Michl, 1962; Michl & Bachmayer, 1963; Bajger, 1980; Simmaco, Kreil & Barra, 2009; Stark & Meiri, 2018). Yet, longevity of some *B. variegata* populations in Germany, Switzerland and Turkey is evidently and consistently less than 10 years being considerably lower than the >20 years in Austria and Italy (Seidel, 1993; Di Cerbo et al., 2011; Bülbül et al., 2018; Cayuela et al., 2019a; Hantzschmann & Sinsch, 2019). The proximate causes for the broad fast–slow continuum in the longevity trait of a toad considered unpalatable are unknown.

In this study, we analyze the among-populations and among-years variation of the life-history traits annual survival and longevity in nine short- and long-lived populations, which represent the fast and the slow extremes of the continuum in Germany and Austria. Long-term Capture-mark-recapture (CMR) monitoring and direct age assessment by skeletochronology allow for reliable estimates of adult survival and longevity. At the local scale, that is, within the area in which the members of a population move, we evaluate the influence of weather, nutritional state and pathogen prevalence on the variation of annual adult survival and the resulting cohort longevity. At the continental scale, that is, the geographical range in which *B. variegata* populations occur, we explore the influence of climate and the allocation of ingested energy to somatic growth on the variation of longevity, supplementing our data with previously published information. We hypothesize that the broad fast–slow continuum of longevity in *B. variegata* is shaped by both an intrinsic feature of each population defining the magnitude of annual survival and by the action of extrinsic mortality factors introducing local variation to annual survival rates. Hence, we generate testable predictions to evaluate this hypothesis: (1) Annual survival rates vary locally among years, but the average magnitude differs among populations. (2) Annual survival rates do not decrease with age within a population (i.e., absence of senescence). (3) The variation of annual survival rate correlates with the among-years variability of local weather and condition index. (4) Longevity variation among populations does not reflect differential energetic investment to somatic growth. (5) Longevity variation among populations is related to climate gradients. (6) Low longevity is associated with high local pathogen prevalence.

**Figure 1:**
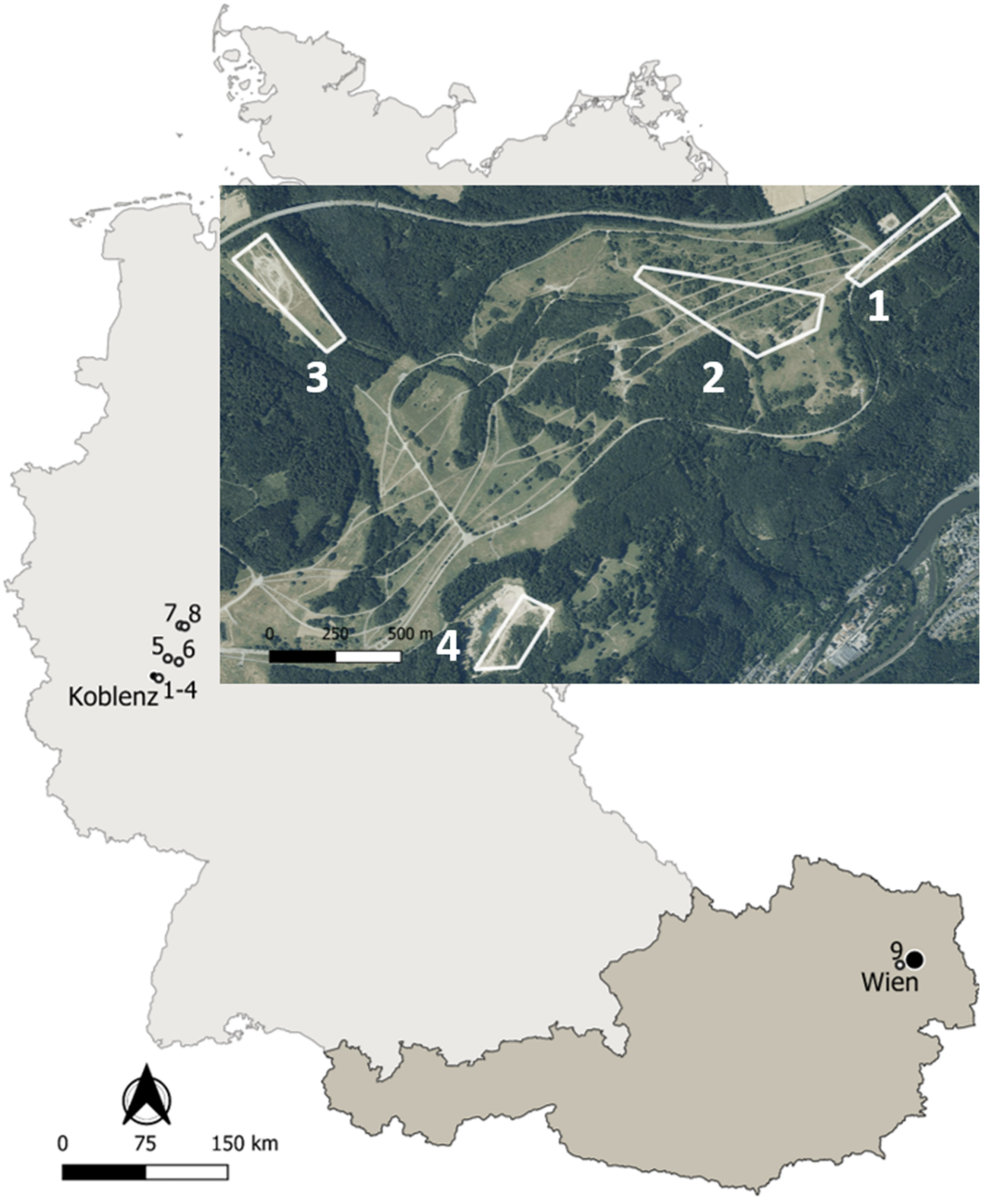
Map of the study sites in Germany and Austria. Details on the localities are available in the text and Table 1. Map edited with the software QGis: http://qgis.osgeo.org; basis data extracted from the GADM database: www.gadm.org; adapted aerial image extracted from the Geoportal RLP: www.geoportal.rlp.de, ©GeoBasis-DE/LVermGeoRP (2019), dl-de/by-2-0, www.lvermgeo.rlp.de.

## Materials and Methods

### Study area

Life-history traits of yellow-bellied toads *Bombina variegata* were studied in nine localities corresponding to populations 1–8 in Germany and to population 9 in Austria (Fig. 1). Details on geographic location, local climate, survey years and sampling frequency are given in Table 1. Climate data were obtained from the nearby weather stations Montabaur (50.44°N, 7.81°E, 265 m asl, Germany), Marienberg (50.66°N, 7.96°E, 547 m asl, Germany) of the Deutscher Wetterdienst, and Mariabrunn (48.21°N, 16.23°E, 225 m asl, Austria) of the Zentralanstalt für Meteorologie und Geodynamik.

Populations 1–3 inhabited the former military training area Schmidtenhöhe (area: ca. 700 ha) and population 4 a neighbouring clay pit near Koblenz (Rhineland-Palatinate, Germany; Hantzschmann & Sinsch, 2019), now forming part of the NATURA 2000 network (LNatSchG, 2015). The localities occupied by populations 1–4 were about 0.5–2.4 km distant from each other. Since we did not detect any exchange of toad individuals among the four areas, we considered the breeding assemblage at each locality as an isolated and independent population. The annual migratory range of *B. variegata* is 20–244 m in Bulgaria (Beshkov & Jameson, 1980), 6–600 m in Romania (Hartel, 2008), 203–732 m in Germany (Jacob, Scheel & Buschmann, 2009), and 300 m in Switzerland (Jordan, 2012) supporting the assumed isolation of populations 1+2, 3 and 4. Populations 5-8 were sampled at working and former clay pits 18–48 km distant from populations 1–4 to supplement longevity estimates based on skeletochronology. Population 9 was located in the north-western part of Lainzer Tiergarten, a nature reserve in the west of Vienna (Austria), which is covered by deciduous forest interspersed with meadows. The area (Hochwiese, ca. 1 km^2^) surveyed for this study consisted of a large wet meadow where small puddles are formed by wallowing wild boars (*Sus scofra*), and three streams in its immediate vicinity.

The Ethics committee of FB3, Department of Biology, University of Koblenz–Landau (#Bv 01/V/2004), approved research. Research and collecting permits referring to the directive #92/43/EWG (FFH-directive) and to the German Federal Law of Nature Conservation (Bundesnaturschutzgesetz, BNatSchG) were issued by the Struktur- und Genehmigungsdirektion Nord in Koblenz (425-104.111.0501; 0603; 0702; 1602; 1711), and by the municipality of Vienna (MA22–2752/96, MA22–1451/06 and MA 22–984143-2015-5) in Austria. All applicable institutional and national guidelines for the care and use of animals were followed (Kreisverwaltung Mayen-Koblenz, Az 39183-04).

**Table 1:** List of study localities, survey periods and number of adult individuals collected per year. Climate data are given as annual average and corresponding range of the local study periods and refer to the stations Montabaur (50.44°N, 7.81°E, 265 m asl), Marienberg (50.66°N, 7.96°E, 547 m asl), and Mariabrunn (48.21°N, 16.23°E, 225 m asl). Raw data on local climate by courtesy of the Deutscher Wetterdienst (Germany) and the Zentralanstalt für Meteorologie und Geodynamik (Austria).

**Population reference number**	**Name and description of study sites**	**Number of ponds (survey area)**	**Latitude [°N], Longitude [°E], Altitude [m asl]**	**Air temperature [°C]**	**Annual precipitation [mm]**	**Study periods**	**Number of sampling surveys**	**Number of adults collected**
**1**	Schmidtenhöhe, vehicle training area,	24 (1.8 ha)	50°20′55″	9.7	787	2004: June 1–July 26	8	31 ♂, 30 ♀
	Germany		7°40′38″	8.3-10.7	589-936	2005: April 21–August 24	19	27 ♂, 43 ♀
			339			2006: April 28–July 23	13	13 ♂, 21 ♀
						2007: April 18–July 18	13	21 ♂, 43 ♀
						2008: April 28–June 12	4	7 ♂, 12 ♀
						2011: May-July	5	6 ♂
						2012: May-July	3	3 ♂
						2016: May 11–September 7	12	17 ♂, 14 ♀
						2017: May 5–July 26	11	16 ♂, 20 ♀
						2018: April 27–August 2	13	26 ♂, 21 ♀
**2**	Schmidtenhöhe, pasture area, Germany	29 (5.8 ha)	50°20′43″	9.7	787	2016: May 17–September 7	16	17 ♂, 4 ♀
			7°40′15″	8.3-10.7	589-936	2017: May 11–July 27	13	43 ♂, 27 ♀
			333			2018: April 27–August 24	16	42 ♂, 29 ♀
**3**	Schmidtenhöhe, tank driving area, Germany	36 (4.2 ha)	50°20′48″	9.7	787	2016: May 26–September 6	16	28 ♂, 27 ♀
			7°38′34″	8.3-10.7	589-936	2017: May 11–July 28	12	44 ♂, 26 ♀
			275			2018: May 5–August 14	10	30 ♂, 20 ♀
**4**	Schmidtenhöhe, clay pit, Germany	35 (3.5 ha)	50°20′07″	9.7	787	2016: May 12–September 7	12	23 ♂, 16 ♀
			7°39′18″	8.3-10.7	589-936	2017: May 6–July 21	12	60 ♂, 50 ♀
			301			2018: April 25–August 24	17	53 ♂, 32 ♀
**5**	Mogendorf, clay pit, Germany	18 (9 ha)	50°29′09″	9.7	787	2017: June 26–July 25	4	21 ♂, 18 ♀
			7°45′41″	8.3-10.7	589-936	2018: July 11 - 12	2	3 ♂, 6 ♀
			294					
**6**	Ruppach-Goldhausen, clay pit, Germany	11 (6.4 ha)	50°27′42″	9.7	787	2017: June 28–July 19	3	28 ♂, 14 ♀
			7°53′20″	8.3-10.7	589-936	2018: July 5 - 11	2	6 ♂, 13 ♀
			255					
**7**	Klebsandgrube Elkenroth, former clay pit,	1 (1.3 ha)	50°44′18″	8.2	1028	2017: June 19–July 24	3	13 ♂, 22 ♀
	Germany		7°43′45″ 443	6.7-9.5	787-1325	2018: July 10	1	2 ♂, 14 ♀
			443					
**8**	Galgenkopf Elkenroth, former clay pit,	9 (0.8 ha)	50°43′23″	8.2	1028	2017: June 22–July 24	3	21 ♂, 12 ♀
	Germany		7°57′18″	6.7-9.5	787-1325	2018: July 10	1	5 ♂, 11 ♀
			451					
**9**	Lainzer Tiergarten, moist meadow, Wien,	10 (15 ha)	48°10′02″	9.9	741	1998: April 22–August 8	7	47 ♂, 45 ♀
	Austria		16°14′36″	9.0-11.2	472-1057	1999: April 14–August 18	7	39 ♂, 36 ♀
			298-315			2000: April 21–September 6	6	57 ♂, 46 ♀
						2001: April 4–October 10	16	67 ♂, 67 ♀
						2002: March 13–September 18	8	62 ♂, 72 ♀
						2003: April 14–September 6	11	72 ♂, 69 ♀
						2004: April 14–October 24	10	72 ♂, 74 ♀
						2005: March 28–October 8	12	71 ♂, 77 ♀
						2006: April 2–October 22	9	72 ♂, 83 ♀
						2007: April 10–September 22	10	67 ♂, 81 ♀
						2008: April 13–September 28	8	71 ♂, 71 ♀
						2009: March 26–September 27	8	69 ♂, 63 ♀
						2010: April 21–September 6	9	68 ♂, 77 ♀
						2011: April 19–September 26	8	80 ♂, 76 ♀
						2012: March 24–October 5	10	72 ♂, 71 ♀
						2013: April 18–September 28	9	74 ♂, 72 ♀
						2014: April 4–September 13	9	71 ♂, 70 ♀
						2015: April 12–October 3	9	67 ♂, 65 ♀
						2016: April 3–October 18	8	46 ♂, 58 ♀
						2017: April 30–October 15	9	32 ♂, 37 ♀
						2018: April 8–September 19	8	50 ♂, 37 ♀

### Field procedures and individual toad identification

At each site surveyed, *B. variegata* specimens were hand-caught at the local ponds and their terrestrial vicinity on a weekly to three-weekly base during the activity period. Capture sessions consisted in an area-constrained, exhaustive search for post-metamorphic individuals without time limit (duration: 0.5 to 5 h). Sex, snout-vent length (SVL, measured with callipers, rounded to the nearest mm), body mass (measured with an electronic balance, rounded to the nearest 100 mg), and ventral spot pattern of each specimen were recorded for individual recognition. Criterion for the distinction among immature, male or female individuals was the minimum SVL of males possessing nuptial pads. Smaller individuals were considered immature, same- or larger-sized individuals without nuptial pads females. In populations 1–8, the photographing procedure involved placing the toads in a petri dish with a lid containing a foam insert (method adapted from Vörös, Szalay & Barabás, 2007; Gollmann & Gollmann, 2011; Cruickshank & Schmidt, 2017). Inverting the petri dish revealed the ventral pattern, which was photographed using different types of digital cameras. In population 9, toads were held in the hand for photography. Additionally, we collected phalange bones from all adult individuals captured in populations 1–8. For age determination, the 3rd or 4th digit of a forelimb was toe-clipped using ethanol-sterilised scissors. We did not sample recaptured adults (i.e., those lacking a digit) again. The wound was cleaned with 70% ethanol and the individual was released *in situ*. The digit was stored in 100% ethanol or in 4% formaldehyde solution at room temperature until skeletochronological laboratory processing.

Handling times were less than one minute per animal, and we released individuals at their original capture location once photographs had been taken. Matching of images to identify recaptured individuals was done by a combination of searching a photographic database with all records using the Wild-ID software (Bolger et al., 2012) and subsequent confirmation of identity of the best 20 potential matches by eye (Cruickshank & Schmidt, 2017). In population 9, identification of individuals was exclusively done by eye, partly in the field using a printed catalogue of ventral patterns (Gollmann & Gollmann, 2012).

### Demographic life-history traits examined

#### Annual recapture/survival rates

Recapture/survival rates of adults and their variation among years were assessed in populations 1–4 (Schmidtenhöhe, Germany) and 9 (Lainzer Tiergarten, Austria). We used Capture-Mark-Recapture (CMR) surveys and stationary life-tables to estimate the annual recapture rates as a proxy of survival at a given locality. We defined all adult individuals independent of actual age that were captured within one year in a given study area as the adult cohort of year x. For each cohort, we constructed a matrix consisting of presence/absence data for the first capture year and subsequent recaptures during the following years. We calculated two measures of the recapture rate: (1) RRR (raw recapture rate) = actually recaptured adults in year x+1/number of adults captured in year x; (2) CRR (corrected recapture rate) = (actually recaptured adults in year x+1 + number of adults of this cohort recaptured during later years)/number of adults captured in year x. The actual annual survival rate was underestimated by the two measures due to the imperfect detection of the cohort members during surveys. Yet, CRR is expected to be a close estimate because of the high number of surveys within each year covering most of the activity period of toads. The number of actually captured toads was 88–94% of the Lincoln-Peterson estimate of population size suggesting a high detection probability.

We obtained an independent estimate of the annual survival rate for populations 1–8 using the skeletochronological age data to construct static life tables for a given locality. Static life tables sample all adults alive during a given year assuming a stationary population with overlapping generations. Static and cohort life tables are identical if the population is in equilibrium and the environment does not change, i.e., birth and death rate do not vary significantly in time and there is no immigration and emigration involved. Long-term monitoring of *B. variegata* populations suggests that they are in a near-stationary state with low fluctuation in adult population size (e.g., Gollmann, 2005; Primus, 2013; Hantzschmann & Sinsch, 2019). Then, the l(x) column of the life table (age-specific survival) gives the stable age distribution directly, the q(x) column the stage-specific (yearly) mortality and 1-q(x) the annual survival rate.

### Statistical analyses

We compared the normally distributed local RRR and CRR estimates following the first year after initial capture using an ANOVA with multiple group comparison by the post-hoc Tukey-HSD test. Since cohort recapture rates decreased in time during the following years at each locality, we tested, if the differences among populations persisted using an ANCOVA with the covariate time (year of recapture − year of initial capture). The effects of toad age (senescence) and study year on CRR were tested for in a 2-factor ANOVA. Calculations were run using the program package Statgraphics Centurion version 18.1.01. The significance level was set at alpha = 0.05.

### Longevity in the surveyed populations

Longevity was assessed in all populations. Estimates were obtained using the CMR data and skeletochronological age determination for each adult cohort separately. CMR data served as a conservative estimate of the lifespan by counting the number of consecutive years in which an adult was recaptured and adding two more years for the post-metamorphic period before sexual maturity (Gollmann & Gollmann, 2012). To obtain an exact estimate, this method requires that the period covered by recapture events is longer than local longevity. This was true for some cohorts of populations 1 and 9. Additionally, we calculated a probabilistic estimate of the minimum lifespan of the eldest 1% adults of a cohort for population 9 in which the recapture period was often shorter than local longevity. We fitted a square-root(x) regression model to the corrected recapture rates [P = a + b*sqrt(x)], where P is the probability that an individual is recaptured x years or more after the first capture. Then, the longevity estimate is recapture year x at *P* = 0.01 plus 2 years for the juvenile period.

Skeletochronological age estimation is based on quantifying the number of dense hematoxylinophilic narrow growth marks (Lines of Arrested Growth, LAGs, each representing a hibernation) separated by faintly stained broad growth zones in the round bones of amphibians (Smirina, 1972). Individuals of up to eight years of age are correctly aged by LAG-counting, whereas the lifespan of older individuals is systematically underestimated, due to the increasing rapprochement of LAGs at the periphery of bones (review: Sinsch, 2015). Standard laboratory protocol included embedding of the phalange samples in Historesin™ (JUNG), cross-sectioning of diaphysis (12 µm) using a JUNG RM2055 rotation microtome, and staining of cross sections with 0.5% cresylviolet. Cross sections were examined under a light microscope for the presence of growth marks at magnifications of 400x using an OLYMPUS BX 50. The first and the senior author assessed the number of LAGs independently. Age is the maximum number of LAGs detected in an individual plus one for the year of capture, i.e., the activity period following the last hibernation. Then, longevity at a given locality is the maximum age that was recorded throughout the whole study period.

### Statistical analyses

We compared the log10-normalized longevity estimates using an ANCOVA with the fixed factor locality and the continuous covariate year. To test for group differences, we used multiple group comparison by the post-hoc Tukey-HSD test (Statgraphics Centurion version 18.1.01, significance level alpha = 0.05).

### Potential impact factors on survival rates and longevity

### Weather (local scale)

Local weather conditions vary between years with cold wet winters and hot dry summers and may affect annual survival rates (Weinbach et al., 2018). To quantify the weather/survival relationship, we retrieved data on air temperature and precipitation from weather stations near to populations 1–4 and 9. We calculated two sets of influence parameters: (1) Annual means of average (T_X_), minimum (T_Min_) and maximum air temperature (T_Max_), and annual sum of precipitation (R_Sum_). (2) Average and maximum summer air temperature (June, July, August; ST_X_ and ST_Max_) and the corresponding sum of precipitation (SR_Sum_) as a surrogate for summer heat and drought suffered during the activity period in the year of first capture. Analogously, average and minimum winter air temperature (December, January, February; WT_X_ and WT_Min_) and the corresponding sum of precipitation (WR_Sum_) served as surrogates of winter severity.

### Statistical analyses

We applied a multiple regression analysis to quantify the effect of each weather data set complemented with the condition index (see below) on the CRR estimates. To obtain the smallest significant model, we used the procedure: backward selection at *F* < 4.0 to include those weather variables into the model, which explained a significant amount of variance observed in the CRR of the age-mixed cohorts (Statgraphics Centurion version 18.1.01, significance level alpha = 0.05).

### Climate (continental scale)

To test for a general pattern underlying the fast–slow continuum of longevity throughout the geographical range, we identified published information on longevity estimates for *B. variegata* basing on CMR or on skeletochronology. CMR studies qualified, if the study period covered at least two successive years and raw data on recapture were included. These criteria were met by Beshkov & Jameson (1980), Gollmann (1981), Kapfberger (1984), Seidel (1988), Seidel (1992), Seidel (1993), Plytycz & Bigaj (1993), Marchand (1993), Plytycz et al. (1996), Sy (1998), Sy & Grosse (1998), Di Cerbo (2001), Gollmann (2005), Dino, Milesi & Di Cerbo (2010), Di Cerbo et al. (2011), Gollmann & Gollmann (2012), Primus (2013), and Cayuela et al. (2019a). Age estimates by skeletochronology are limited to Seidel (1988), Plytycz & Bigaj (1993), and Bülbül et al. (2018). Longevity data were obtained for 21 localities including our own study sites (Tables 1 and 2). As surrogate measures of local climate, we used the combinations of (1) latitude, longitude and altitude (coarse-grained approach), and of (2) average annual air temperature and annual sum of precipitation (fine-grained approach). To obtain empirically measured data on temperature and rainfall, we retrieved data from nearby weather stations (within a radius of 20 km) at a similar altitude (within a range of ±100 m asl) as that of the study locality during the study periods at each site.

**Table 2:** Published information on longevity of *B. variegata* throughout the geographical range. Estimates given in parentheses are based on CMR-studies covering less than four successive years.

**Study sites**	**Latitude[°N]**	**Longitude[°E]**	**Altitude[m asl]**	**Study period**	**Estimated maximum lifespan**		**Reference**
					**CMR [years between first and last capture + 2]**	**Skeletochronology[n LAGs + 1]**	
Ballertasche, Hannoverisch Münden, Germany	51°27′29″	9°38′13″	122	1986-1989	(5)	–	Marchand (1993)
Dörnaer Platz, Mühlhausen, Germany	51°13′23″	10°23′30″	300-360	1988-1992	11	–	Sy (1998), Sy & Grosse (1998)
Siegelsdorf, Fürth, Germany	49°30′12″	10°52′11″	327	1978-1981	(5)	–	Kapfberger (1984)
Sądecka Mountains, Jasieńczyk, Poland	49°35′56″	20°23′28″	600-630	1981-1994	15	12	Plytycz & Bigaj (1993), Plytycz et al. (1996)
Ottenstein/Dobra, Austria	48°35′45″	15°20′22″	440-530	1974, 1979-1980, 1984-1991	15	8	Gollmann (1981), Seidel (1988), Seidel (1992), Seidel (1993)
Klausen-Leopoldsdorf, Großkrotten-bach, Austria	48°05′18″	16°00′54″	374	2002-2011	11	–	Gollmann (2005), Primus (2013)
Schwyz, stable habitat, Switzerland	47°01′05″	8°36′00″	450	2011–2015	4.8^*^	–	Cayuela et al. (2019a)
Schwyz, instable habitat, Switzerland	47°01′05″	8°36′00″	450	2011–2015	13.5^*^	–	Cayuela et al. (2019a)
Albino, Italy	45°46′07″	9°47′05″	450	1994-2010	20	–	Di Cerbo (2001), Dino, Milesi & Di Cerbo (2010), Di Cerbo et al. (2011)
Parco dei Colli di Bergamo, Italy	45°48′30″	9°47′12″	1148	1988-2010	20	–	Di Cerbo (2001), Dino, Milesi & Di Cerbo (2010), Di Cerbo et al. (2011)
Manastirishte, Buchino Prohod	42°58′31″	23°08′19″	780-820	1977-1978	(4)	–	Beshkov & Jameson (1980)
Enez, Edirne	40°45′10″	26°13′51″	181	2016	–	9	Bülbül et al. (2018)

**Notes.**

* Longevity estimates given by Cayuela et al. (2019a) were calculated using multievent models.

### Statistical analyses

We used a multiple regression analysis to assess the correlation between log10-normalized longevity and log10-transformed data sets 1 and 2. Power analyses showed that 21 observations allowed for the detection of significant multiple correlation at *R*^2^ = 0.51 (data set 1) and *R*^2^ = 0.47 (data set 2), respectively. As study periods reported were short in several case studies, we cannot exclude that published longevity does not represent precisely actual maximum life expectancy. Therefore, we used a supplemental discriminant analysis to distinguish the geographical features of localities inhabited by short-lived (reported longevity less than 10 years) or long-lived populations (reported longevity at least 20 years), i.e., the extremes of the fast–slow continuum. Multiple regression and discriminant analyses were performed using the program package Statgraphics Centurion version 18.1.01, power analyses using G*Power version 3.1.9.2. (Faul et al., 2009). The significance level was set at alpha = 0.05.

### Energetic investment to somatic growth and to reserves for winter survival

The allocation of ingested energy to somatic growth and subsequently, the growth pattern (age-size relationship) depend on food availability in the habitat and has consequences for longevity (Kozłowski, 1992; Sztatecsny & Schabetsberger, 2005; Hecht et al., 2019). We quantified the seasonal variation of the condition index as a surrogate measure of the nutritional state at capture for populations 1–4 and 9. The growth patterns of populations 1–9 reflect the long-term investment of food to somatic growth. The condition index of an individual is calculated as the studentized residual of the SVL-mass relationship using a multiplicative model ln(mass) = a + b × ln(SVL), with a = intercept and b = slope (residual index: Băncilă et al., 2010; Scheele et al., 2014; Mikoláš, 2016). Growth following metamorphosis was estimated using the Von Bertalanffy equation (1938): SVL_t_ = SVL_max_ − (SVL_max_ − SVL_met_) * e^−k∗t^, where SVL_t_ = average body length at age t; SVL_max_ = asymptotic body length; SVL_met_ = body length at metamorphosis; t = number of growing seasons experienced (n LAGs), and k = growth coefficient (i.e., shape of the growth curve). SVL_met_ varied between 11 mm and 19 mm (Schäfer et al., 2018) and was set to an average value of 15 mm (Di Cerbo & Biancardi, 2010). We analysed age/size data for populations 1–8 based on skeletochronological estimates, and for population 9 based on CMR age data.

### Statistical analyses

To detect differences in the nutritional state of toads at the study sites, we analysed the variation of the condition index in a 2-Factor ANCOVA with sex and locality as categorical factors, and activity season (March 26 to October 10, i.e., day 85 to 283) as continuous co-variable. The von Bertalanffy growth model was fitted to the average growth curve using the least square procedure (nonlinear regression). Estimates of SVL_max_ and k are given with the corresponding 95% confidence interval. If confidence intervals did not overlap in pairwise comparisons, estimates differed significantly at *P* < 0.05. Statistical procedures were performed using the program package Statgraphics Centurion version 18.1.01.

### Pathogen prevalence in short-lived populations

Disease- and parasite-mediated reduction of longevity may affect *B. variegata* populations (Spitzen-van der Sluijs et al., 2017; Campbell et al., 2018). Therefore, we looked for dead specimens and living individuals showing external signs of disease such as skin lesions, ectoparasites, and abnormal behaviour in populations 1–8. Dead individuals, which were not run over by tanks or cars or with marks of predator attack, were dissected to assess the presence of endoparasites (Sinsch, Kaschek & Wiebe, 2018). The skin and nostrils of *Bombina* were examined for the presence of eggs and larvae of the blowfly *Lucilia bufonivora* Moniez, 1876, infecting common toads *Bufo bufo* at the Schmidtenhöhe. In 2017, we sent phalange samples of 49 individuals collected in populations 1–4 to the Zoological Society of London (Institute of Zoology) to detect infections with the chytrid fungus *Batrachochytrium dendrobatidis* and Ranavirus.

## Results

**Figure 2:**
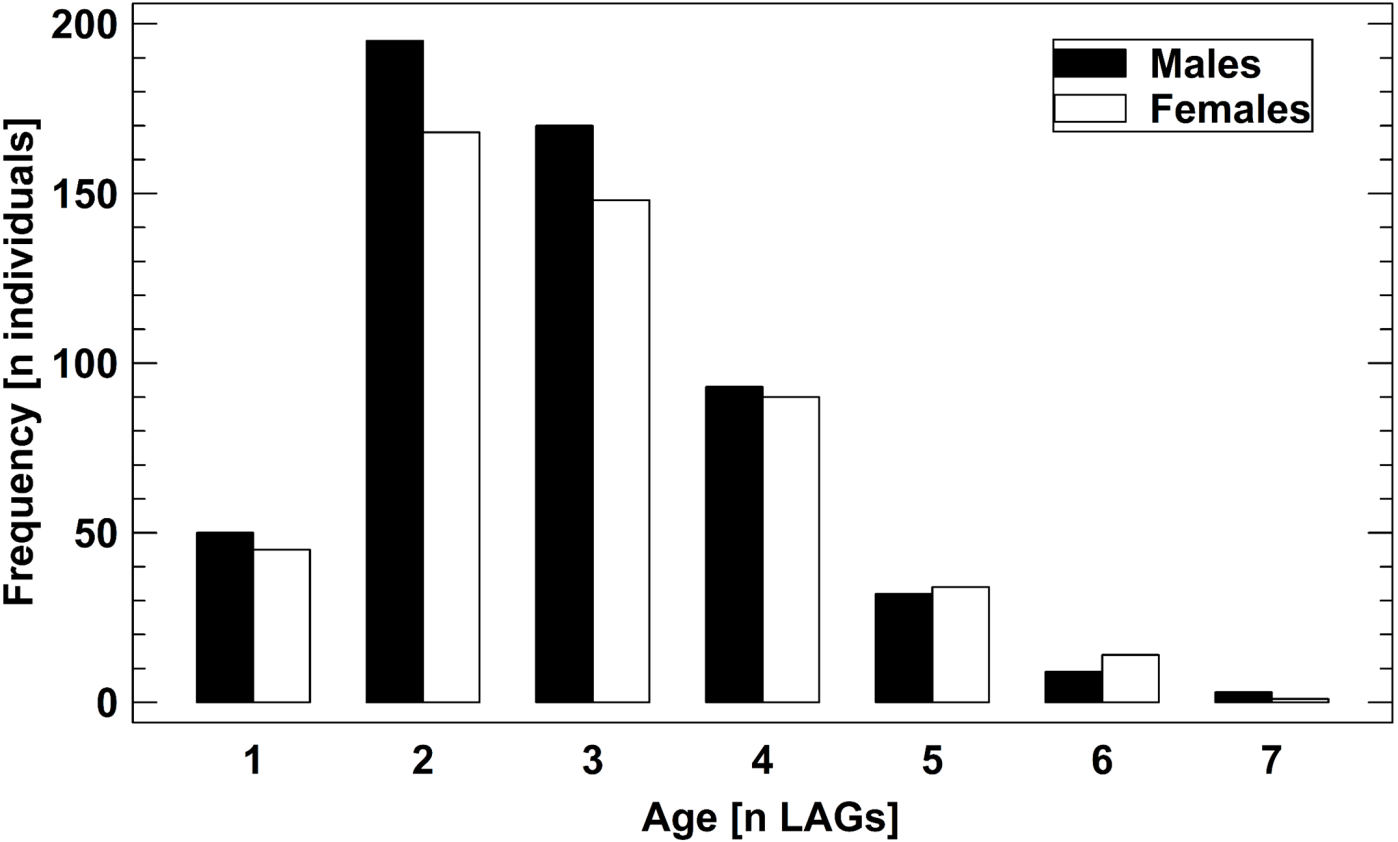
Age structure of 1,061 adult *B. variegata* in the Westerwald region. Data are pooled from all study periods and include populations 1–8 (Fig. S1, Table 1). Log10-transformed age did not differ between males and females (2-factor ANOVA: *F*_1,1060_ = 1.60, *P* = 0.2059) and among populations (2-factor ANOVA: *F*_7,1060_ = 1.41, *P* = 0.1958).

The age structure of male and female *B. variegata* did not differ significantly (Fig. 2, Fig. S1), as suggested by an earlier study (Bülbül et al., 2018). Therefore, we pooled data on survival and longevity irrespective of gender for further analyses. Life-history traits studied in nine populations are summarized in Table 3. Results are presented in the order of the predictions tested.

**Table 3:** Life-history traits of *B. variegata* at the study sites.

**Population**	**CRR**	**Longevity[years]**	**Condition index**	**Growth pattern**	
				**SVL**_**max**_**[mm]**	**k**
**1**	0.30 ^a^ (0.18–0.42)	5-8	−0.46 ^a^ (-0.56/-0.036)	50.0 ^a^ (48.1–51.9)	0.442 ^a^ (0.397-0.488)
**2**	0.52 ^a^ (0.41–0.63)	5-7	0.28 ^d^ (+0.16/+0.40)	49.0 ^a^ (45.7–52.3)	0.424 ^a^ (0.342-0.505)
**3**	0.55 ^a,b^ (0.41–0.69)	5-8	0.08 ^c^ (0.00/+0.16)	50.5 ^a^ (48.0–52.9)	0.370 ^a^ (0.328-0.413)
**4**	0.49 ^a^ (0.38–0.60)	6-7	0.39 ^d^ (0.27/+0.51)	47.3 ^a^ (44.4–50.1)	0.484 ^a^ (0.399-0.570)
**5**	–	5-8	–	46.7 ^a^ (43.8–49.5)	0.470 ^a^ (0.373-0.567)
**6**	–	5-6	–	49.2 ^a^ (43.6–54.8)	0.422 ^a^ (0.302-0.542)
**7**	–	6-8	–	46.6 ^a^ (43.8–49.4)	0.566 ^a^ (0.473-0.657)
**8**	–	5-8	–	47.5 ^a^ (44.6–50.3)	0.444 ^a^ (0.399-0.610)
**9**	0.71 ^b^ (0.65–0.77)	13-23	−0.06 ^b^ (-0.16/+0.04)	50.6 ^a^ (49.4–51.8)	0.433 ^a^ (0.392-0.474)

**Notes.**

CRR Corrected recapture rate longevity lifespan of the oldest individual detected by CMR or skeletochronology Condition index studentized residual of age-mass regression growth pattern asymptotic maximum SVL and growth coefficient of von Bertalanffy growth models

Except for longevity (range of annual estimates), data are given as average and corresponding 95% confidence interval. Hyphenated letters indicate significant difference among populations (*P* < 0.05). For statistical details see text.

### Prediction 1: Annual survival rates vary locally among years, but the average magnitude differs among populations

We compared recapture rates (X ± SE) of age-mixed adult populations one year following the first capture among populations 1–4 (Schmidtenhöhe, Germany) and 9 (Lainzer Tiergarten, Austria) to quantify the variation of the corresponding survival rates. Recapture rates differed significantly among populations 1–4 (ANOVA: RRR, *F*_4,31_ = 7.25, *P* = 0.0004; CRR, *F*_4,25_ = 10.49, *P* = 0.0001). A closer inspection showed that population 9 differed from all other populations (Tukey-HSD test, *P* < 0.05). Average RRR were 0.32 ± 0.04 (populations 1–4) and 0.52 ± 0.03 (population 9), corresponding average CRR were 0.41 ± 0.06 and 0.71 ±0.03.

**Figure 3:**
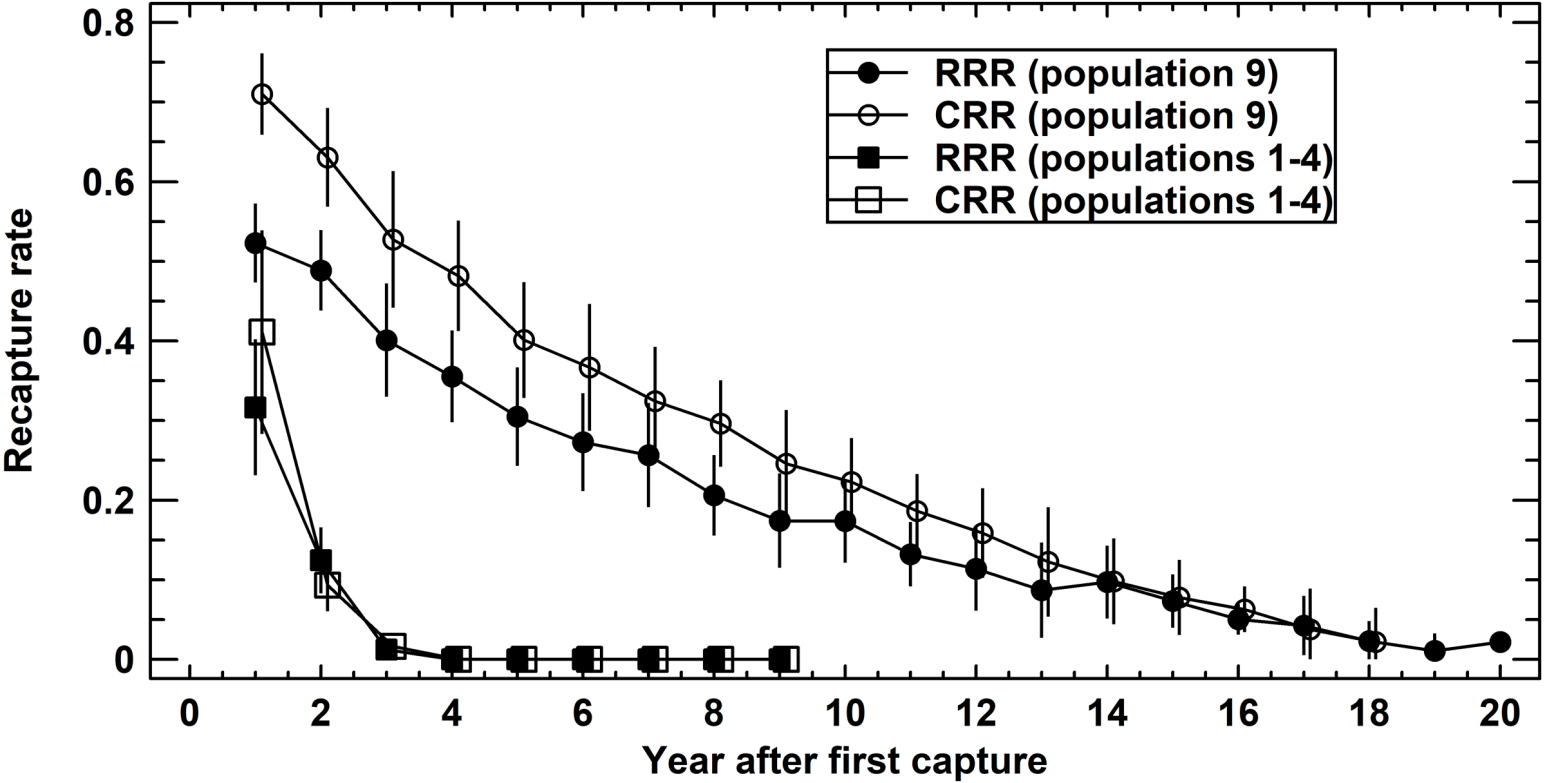
Recapture rates in Germany (populations 1–4 pooled) and Austria (population 9). Raw recapture rates (RRR, solid symbols) consider exclusively the actually recaptured adults per year. Corrected recapture rates (CRR, open symbols) also include those adults known to be alive in the reference year, but that were not recaptured. For details see text.

When using the number of years after first capture as a covariate of recapture rates (ANCOVA: RRR, *F*_1,250_ = 397.4, *P* < 0.0001; CRR, *F*_1,237_ = 614.0, *P* < 0.0001), the difference persisted (ANCOVA: RRR, *F*_1,250_ = 178.6, *P* < 0.0001; CRR, *F*_1,237_ = 177.4, *P* < 0.0001) (Fig. 3). Comparing RRR and CRR at each locality, again using the number of years after first capture as a covariate, CRR was significantly greater than RRR in population 9 (ANCOVA: *F*_1,398_ = 52.0, *P* < 0.0001), but not in populations 1–4 (ANCOVA: *F*_1,67_ = 1.3, *P* = 0.2611). In summary, annual survival rates varied within each population, but differed significantly in magnitude between the four short-lived populations and the long-lived one.

### Prediction 2: Annual survival rates do not decrease with age within a population

**Figure 4:**
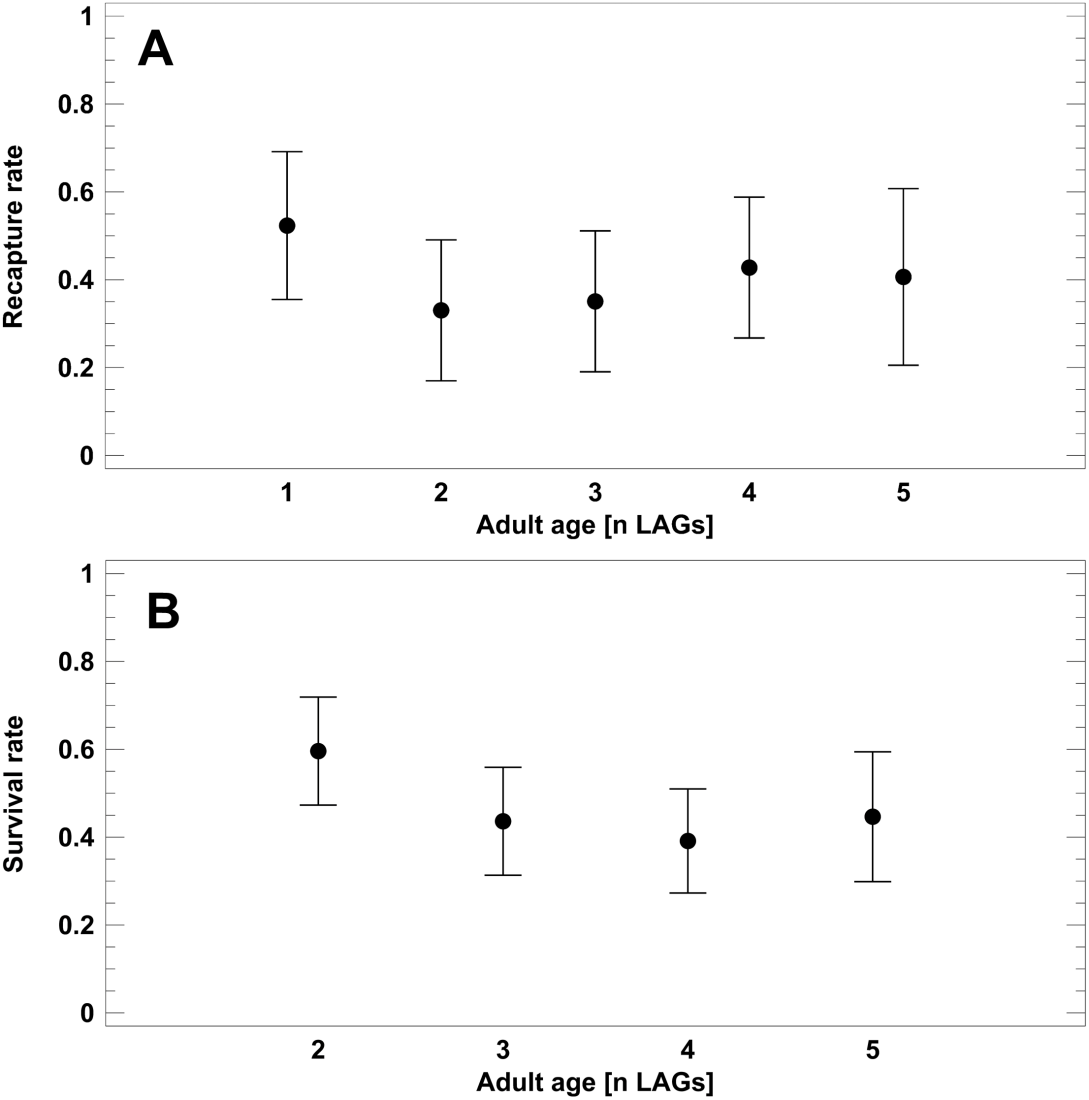
Corrected recapture rates (CRR) of known-age adult classes (A) and survival rates estimated from static life tables (B). Analyses refer exclusively to the *B. variegata* populations 1–4 (Schmidtenhöhe, Germany). Age is given as number of LAGs. Data are given as average (dots) and corresponding 95% Tukey-HSD confidence intervals.

To test for senescence in populations 1–4 (Schmidtenhöhe, Germany), we analysed recapture rates of known-age toads, i.e., skeletochronologically studied individuals. Corrected recapture rate did not differ among the age classes (1–5 LAGs; 2-factor ANOVA: *F*_4,49_ = 1.4, *P* = 0.2687; Fig. 4A), but varied among the survey years (2-factor ANOVA: *F*_4,49_ = 3.2, *P* = 0.0223). Survival to the next year was significantly higher in 2016 (CRR=0.58) than in 2005 (0.26) and 2017 (0.33; Tukey-HSD test, *P* < 0.05). Average recapture rate (X ± SE) of known-age adults was 0.38 ± 0.04. Survival rates (SR) estimated from the static life tables did not differ significantly among the main adult age classes (Fig. 4B). Average survival rate was 0.46 ± 0.03. Estimated annual survival rates tended to be slightly higher on average than corresponding CRR, but the 95% confidence intervals overlapped widely: 0.40–0.52 (SR) *vs.* 0.31- 0.52 (CRR). In summary, we did not detect senescence in the short-lived populations.

### Prediction 3: The variation of annual survival rate correlates with the among-years variability of local weather and condition index

In populations 1–4 (Schmidtenhöhe, Germany), the most explanatory regression model based on yearly averages of temperature descriptors and of precipitation regime explained 63.4% (R^2^ corrected for df) of the among-years variation of CRR (Multiple Regression Analysis: *F*_2,11_ = 10.51, *P* = 0.0044). The model included only the average annual air temperature T_X_ and average annual maximum of daily air temperature T_Max_, whereas precipitation and condition index did not contribute significantly to CRR variation. Using descriptors of winter and summer conditions instead of yearly averages, local weather variation accounted almost entirely for the corresponding variation of CRR (Multiple Regression Analysis: R^2^(corrected for df)=90.7%, *F*_5,11_ = 22.56, *P* = 0.0008). The refined model included five weather variables following the equation CRR = -0.165 - 0.209*WT_Min_ + 0.009*WR_Sum_ –0.605*ST_X_ + 0.216*ST_Max_ –0.002*SR_Sum_. Again, condition index did not account significantly for CRR variation. Cold winters and warm summers affected CRR positive, whereas precipitation was of minor importance (Fig. S2). In contrast, variation of weather at population 9 (Lainzer Tiergarten, Austria) did not account for a significant amount of among-years variation in CRR as none of the weather variables correlated significantly (Regression analyses, *P* > 0.05). Overall, weather regime at the Lainzer Tiergarten (Austria) was more continental than that at the Schmidtenhöhe (Germany), i.e., minima during winter were lower and average summer temperature was higher (Fig. S2).

### Prediction 4: Longevity variation among populations does not reflect differential energetic investment to somatic growth

Longevity of age-mixed cohorts varied in all populations among years (Table 3). In the short-lived populations 1–8, variation was from 5 to 8 years (skeletochronological and CMR data), in the long-lived population 9 (CMR data, probabilistic estimate for the cohorts 1998-2008) from 13 to 23 years. In May 2019, the recapture of a female of the 1998 cohort (SVL_1998_ = 47 mm) in population 9 provided the first empiric evidence for a realized 23-years longevity. Log10-transformed longevity estimates differed significantly among populations (ANCOVA, *F*_8,35_ = 10.2, *P* < 0.0001), which formed one homogeneous group including populations 1–8 and another represented by population 9 (Tukey HSD-test, *P* < 0.05). The covariate study year was marginally significant (ANCOVA, *F*_1,35_ = 4.34, *P* = 0.0471) showing a slight trend of decreasing longevity during the study period.

The condition index (descriptor of nutritional state) did not differ between males and females (2-Factor ANCOVA, *F*_1,1956_ = 0.48, *P* = 0.4863) and its seasonal fluctuations between spring and autumn did not co-vary with time (2-Factor ANCOVA, *F*_1,1956_ = 1.55, *P* = 0.2126). In contrast, there were significant differences among the populations (2-Factor ANCOVA, *F*_4,1956_ = 41.35, *P* < 0.0001; Table 3). The distinction between short- and long-lived populations by annual survival rate and longevity did not correspond to the complex pattern of condition index variation (Table 3).

The age-SVL relationship (descriptor of long-term investment to somatic growth) did not differ significantly in SVL_max_ or k among short- and long-lived populations (Table 3; Fig. S3). The asymptotic maximum SVL ranged from 46.6 mm to 50.6 mm, the growth coefficient k was similar in all populations. The recalculated growth pattern (model parameters: SVL_max_ = 52.2 mm [47.5–56.9 mm], *k* = 0.327 [0.235–0.419]) of the short-lived population at Enez, Turkey (Bülbül et al., 2018), did not differ significantly from the populations studied by us. In summary, the invested energy to somatic growth was similar from Germany to Turkey, and in short- and long-lived populations.

### Prediction 5: Longevity variation among populations is related to climate gradients

Longevity estimates demonstrated a fast–slow continuum of populations throughout the geographical range (Tables 2 and 3). In 13 populations (10 in Germany, 1 in Bulgaria, 1 in Switzerland, 1 in Turkey), *B. variegata* were short-lived, i.e., longevity estimates ranged from 5 to 9 years. In three populations (1 in Austria, 2 in Italy), toads were long-lived with a realized longevity of 20 to 23 years. In-between these extremes of the continuum, we detected five populations (1 in Germany, 1 in Switzerland, 2 in Austria, 1 in Poland) with a longevity ranging from 11 to 15 years, partially overlapping with the within-population range of longevity variation in population 9 (Lainzer Tiergarten, Austria). The log10-transformed maximum longevity estimate per locality (*n* = 21) did not correlate significantly with local geographical features (multiple regression analysis: *R*^2^ = 0.137, *F*_3,20_ = 0.9, *P* = 0.4630), or with local temperature and rainfall regime (multiple regression analysis: *R*^2^ = 0.199, *F*_2,20_ = 2.25, *P* = 0.1346). When the separate cohort estimates for populations 1–9 were included, log10-transformed longevity correlated significantly with local geographical features (multiple regression analysis: *R*^2^ = 0.266, *F*_3,47_ = 6.68, *P* = 0.0008), and also with local temperature and rainfall regime (multiple regression analysis: *R*^2^ = 0.115, *F*_2,47_ = 4.05, *P* = 0.0247). The presumptive significance is an effect of pseudo-replication that disappears, if we consider exclusively the number of localities for the degrees of freedom. As the calculated multiple correlations were all below the detection thresholds for significance (power analyses), we cannot exclude that local climate accounts for up to 26% of longevity variation. Geographical features (data set 1) and temperature and rainfall regimes (data set 2) of the localities inhabited by short- or long-lived population, did not distinguish between the two ends of the fast–slow continuum (discriminant functions: data set 1, Eigenwert = 0.229, Wilks-Lambda = 0.813, Chi-squared = 2.6; *df* = 3, *P* = 0.4608; data set 2, Eigenwert = 0.378, Wilks-Lambda = 0.726, Chi-squared = 4.2; *df* = 2, *P* = 0.1242).

### Prediction 6: Low longevity is associated with high pathogen prevalence

In the short-lived populations 1–8 (Westerwald, Germany), dead specimens were rarely encountered in the field and with the exception of one individual, all (*n* = 5, 2004-2018) were run over by tanks or cars. A dead female of population 3 found at the shore of a pond in 2017 and without external lesions was checked for endoparasites. The digestive system contained a 35 mm *Megalobatrachonema terdentatum* female (Nematoda). Mass die-offs of *B. variegata* or of any syntopic amphibians were never observed. Blowfly (*Lucilia bufonivora*) eggs were found on the skin of a single female of population 3 in 2016. Indication of a former infection was detected in form of dark spots at the nostrils of another female of population 4 in the same year. There was evidence for the presence of *Batrachochytrium dendrobatidis* in 6 individuals of populations 1 and 2, and by *Ranavirus* in one individual of population 2. In summary, pathogens in low prevalence were present in most of the short-lived populations studied.

## Discussion

Our case study on *B. variegata* provides evidence that the magnitude of longevity is a fixed attribute of a population, which varies among the cohorts of a population by integrating the influence of environmental stochasticity on annual survival rates during the cohorts’ lifetime. The range of variation was up to 3 years in short-lived populations and up to 10 years in the long-lived one being considerable smaller than that of the entire specific fast–slow continuum with 18 years (Tables 2 and 3). This pattern of distinctive variation at local and continental scale contrasts with that in *Capensibufo rosei* where the variation of cohort longevity represents the entire fast-slow continuum (Becker et al., 2018). Our data support the hypothesis that longevity variation in *B. variegata* populations at continental scale integrates two components, one defining the magnitude of longevity (intrinsic component) and the second local variation (extrinsic component reflecting environmental stochasticity). Therefore, we focus discussion on the identification of factors acting on each component.

### Sources of local variation in survival rates and longevity

All populations studied varied with respect to these life-history traits confirming predictions 1 and 4. Senescence, i.e., age-specific mortality and the timing of its increase, may be a source of variation in survival rates, as postulated by current theories of aging (antagonistic pleiotropy, disposable soma; Williams, 1957; Kirkwood, 1977; Kirkwood & Austad, 2000). While there is growing evidence for these theories in species with determinate growth (birds and mammals), our data demonstrate that in the short-lived populations the annual survival rate is independent of age. In the long-lived population, the annual survival rate remains high during the entire lifetime of a cohort suggesting that there is no decrease of survival with increasing age. In agreement with a study on palearctic salamanders (Cayuela et al., 2019b), we conclude that senescence is negligible in the variation of annual survival rates confirming prediction 2.

Local temperature and precipitation regimes, i.e., weather, show great among-years variation and may affect survival by affecting water balance and metabolism of amphibians (Hillman et al., 2008). In the short-lived populations of *B. variegata*, weather variables accounted indeed for almost all of the variability in annual survival rates indicating that duration of the activity period (mild winters, Fig. S2A) and temperature-modulated intensity of activity (cold summers, Fig. S2B) play a major role for survival. This is in agreement with prediction 3 and with variation of survival rates in *Capensibufo rosei* (Becker et al., 2018). Still, survival rates never reached the level of that in the long-lived population indicating the presence of an intrinsic longevity component defining the overall magnitude of survival. In this population, variation of annual survival rates was unrelated to local weather variability suggesting that major mortality factors differ in nature between localities. For example, Cayuela et al. (2017) propose that antipredator and antipathogen skin secretions and modification of reproduction behaviour, i.e., skipping reproduction under harsh conditions, increase adult survival in a French population of *B. variegata*. With respect to global climate change, the marginally significant trend to decreasing longevity in our study populations during the past 20 years may suggest the presence of an additional source of variation. Yet, database is still too poor to lean hard evidence to this trend. If the trend proves true, prolongation of the activity period and corresponding longer exposure to predators may underlie the putative reduction of longevity.

The nutritional state is related to the availability and quality of food in the toads’ habitat and determines the availability of energy reserves for metabolism during hibernation and subsequently survival (Hillman et al., 2008). Using the condition index as a rough estimate for nutritional state and reserves (mass contribution of fat body), we did not detect a seasonal change during the activity period at any study site. If the condition index does reflect the amount of fat body (Hecht et al., 2019), metabolic reserves were probably not depleted substantially during winter or increased to the end of the activity season to prepare for hibernation. As the condition index did not correlate with annual survival rates as well, we conclude that the nutritional state of toads was probably not a major source of mortality. Alternatively, the momentary condition index may be too rough to mirror correctly nutritional state in *B. variegata*.

If there is a trade-off between allocation of resources to reproduction in early life and somatic maintenance (disposable soma theory of aging; Kirkwood, 1977; Kirkwood & Austad, 2000; Räsänen et al., 2008), we expect that the long-term investment into somatic growth should lead to distinct growth pattern in short- and long-lived populations. Yet, we do not find any evidence that toads of long-lived population allocate more resources to somatic maintenance/growth than do toads of short-lived population. Contrary to the predictions of life-history theory, we find that age at sexual maturity is about be same at continental scale. Since sexual maturation requires a threshold SVL of about 30–32 mm, the age of first reproduction is usually the third year of life, exceptionally the second (Gollmann & Gollmann, 2012; Bülbül et al., 2018; this study). In conclusion, local growth pattern seems to be invariant throughout the range of distribution confirming prediction 4.

Pathogen prevalence may considerably affect survival and cause local extinction (Adams, Pessier & Briggs, 2017; Finnerty, Shine & Brown, 2018). For example, infections with *Batrachochytrium dendrobatidis* and Ranavirus decrease longevity of Palearctic amphibians (Spitzen-van der Sluijs et al., 2017; Campbell et al., 2018). Despite the occasional detection of *Bd* and Ranavirus in few individuals of the short-lived populations, pathogen prevalence seems too low to indicate substantial disease-driven mortality. Data on parasites are too scarce to be conclusive. Consequently, we have to leave open a possible contribution of pathogens to longevity variation leaving prediction 6 unresolved.

Among the sources of local variation in survival and longevity of *B. variegata*, the only extrinsic factor identified is local weather variability in the short-lived populations, explaining trait variance almost entirely. In contrast, none of factors evaluated turned out to influence variation in the long-lived population. An additional role of climate change in constraining longevity remains unresolved at present.

### Sources of continental variation in longevity

At the continental scale, altitudinal and latitudinal clines in demographic life-history traits are an often-observed pattern in amphibians (Morrison & Hero, 2003; Morrison, Hero & Browning, 2004). Palearctic examples are the widespread anurans *Rana temporaria* (Sinsch, Pelster & Ludwig, 2015) and *Epidalea calamita* (Leskovar et al., 2006; Oromi, Sanuy & Sinsch, 2012). Yet, our study demonstrates that the contribution of geographic clines and climate features to the variance of the fast–slow longevity continuum in *B. variegata* populations is less than 26%, if there is any. Altitude seems to play an insignificant role as exemplified in the Italian populations (Albino, Parco dei Colli di Bergamo; Table 2). In the two populations, longevity is the same (20 years; Di Cerbo et al., 2011) despite of inhabiting habitats at 450 and 1,148 m asl, respectively. We conclude that general climate clines related to latitude and altitude of habitats do not contribute substantially to the marked differences in realized longevity in this species, falsifying prediction 5.

Excluding geographical clines, local climate, resource allocation pattern and pathogens as determinants for the nature of the component determining the magnitude of longevity, i.e., the position of a population within the fast–slow continuum of longevity, the question remains, which factors enable some populations to reach more than 20 years of life expectancy? The key to an answer is probably the fact that longevity in the field reaches almost the life expectancy of captive individuals (Mertens, 1964; Mertens, 1970) indicating that predation plays a minor role in long-lived populations. If we assume that there are no dramatic local differences in predator abundance, we expect that improved antipredator protection of the local toads reduces mortality caused by predators. Skin toxins as chemical defences of amphibians are well known to determine unpalatability, to decrease predation, and to increase survival (Darst, Cummings & Cannatella, 2006; Kowalski et al., 2018; Hettyey et al., 2019).

To stimulate future research, we present here a new hypothesis that can explain the broad range of among-population variation in longevity—the palatability hypothesis. Skin secretions of *B. variegata* include a cocktail of more or less bioactive peptides such as Bombesin, Bombinins and Bradykinin (Simmaco, Kreil & Barra, 2009). Besides anti-bacterial properties, the main effect of these peptides seems to be an interference with the hormonal system of the predator, as insulin-, gastrin- and TRH-releasing properties in mammals and birds have been evidenced (e.g., Marenah et al., 2004; Gonzalez et al., 2008; Simmaco, Kreil & Barra, 2009; Xu & Lai, 2015). Skin peptides of *B. variegata* are apparently not toxic in a sense of causing death to predators but probably have disruptive metabolic effects combined with irritation of mucosa. If these effects are uncomfortable enough for the predator, it will learn to avoid this prey. The palatability hypothesis proposes that the antipredator protection by bioactive skin secretions varies among *B. variegata* populations determining their position in the fast–slow continuum of longevity. If so, longevity variation reflects mainly the effect of predation on more or less chemically protected toads.

Investigations on the quantity of bioactive substances in the skin secretions of *B. variegata* are still lacking. Yet, there is ample evidence that the concentrations of Bradykinin in *Bombina pachypus*, of Bufogenins in *Bufo gargarizans* and of Tetrodoxins in *Notophthalmus viridescens* vary considerably among conspecific populations (Zhang et al., 2005; Yotsu-Yamashita et al., 2012; Ma et al., 2018; Coppari et al., 2019). Multiple, still unknown factors may cause this variation, from environmental variability to differences in the local gene pool (Bókony et al., 2019). Future measurements of skin peptide profiles and their biological activity on potential predators will show, whether there are significant differences among individuals of short- and long-lived populations.

## Conclusions

Our study supports the hypothesis that a population’s position in the fast–slow continuum of longevity is the result of a widely fixed magnitude of longevity (intrinsic factor) which varies within a limited range related to environmental stochasticity (extrinsic factors). Extrinsic factors describing local climate (latitude, altitude, and longitude) and resource allocation to somatic maintenance were evidently unrelated to the magnitude of a population’s longevity. Therefore, we propose that the intrinsic factor determining longevity is the composition and concentration of bioactive skin secretions (palatability hypothesis) which modulates the risk of being preyed on. Local weather variability is the proximate cause of longevity variation within short-lived populations, whereas the source of variation within long-lived populations remains unidentified. The intraspecific fast–slow continuum of longevity in *B. variegata* reflects probably the efficiency of chemical antipredator protection.

##  Supplemental Information

10.7717/peerj.8233/supp-1 Figure S1 Local age structure of adults collected from eight populations in the Westerwald region Data are pooled from all study periods at each locality (for details see Table 1).

10.7717/peerj.8233/supp-2 Figure S2 Weather-associated variation of CRR of age-mixed cohorts in populations 1-4 (Schmidtenhöhe, Germany) and population 9 (Lainzer Tiergarten, Austria) CRR versus (A) the lowest recorded air temperature during winter (December-February), and (B) the average air temperature during summer (June–August).

10.7717/peerj.8233/supp-3 Figure S3 Von Bertalanffy models for the age-SVL relationship in short- and long-lived populations (A) Populations 1-8, Germany (model parameters: SVL_max= 48.6 mm [47.6-49.7 mm], k=0.442 [0.417-0.469]). Data are pooled from all study periods. (B) Population 9, Austria (model parameters: SVL_max= 50.6 mm [49.4-51.8 mm], k=0.433 [0.392-0.474]).

10.7717/peerj.8233/supp-4 Data S1 Age-size raw data Locality, year of capture, sex, SVL and age of individuals from the 9 study sites.

## Additional Information and Declarations

### Field Study Permissions

The following information was supplied relating to field study approvals (i.e., approving body and any reference numbers): In Germany: Research and collecting permits referring to the directive #92/43/EWG (FFH-directive) and to the Federal Law of Nature Conservation (Bundesnaturschutzgesetz, BNatSchG) in Germany were issued by the Struktur- und Genehmigungsdirektion Nord in Koblenz (425-104.111.0501; 0603; 0702; 1602; 1711). In Austria: Capture and handling of the toads were allowed by the municipality of Vienna (MA22–2752/96, MA22–1451/06 and MA 22–984143-2015-5).

### Data Availability

The following information was supplied regarding data availability: Raw data in the Supplemental Information includes the age-size data of toads captured in the nine populations subject to this study.
