# Supplementary figures and images for: The fast–slow continuum of longevity among yellow-bellied toad populations (Bombina variegata): intrinsic and extrinsic drivers of variation
Source: PeerJ. 2019 Dec 16;7:e8233. doi: 10.7717/peerj.8233 (PMC6921980; doi:10.7717/peerj.8233)

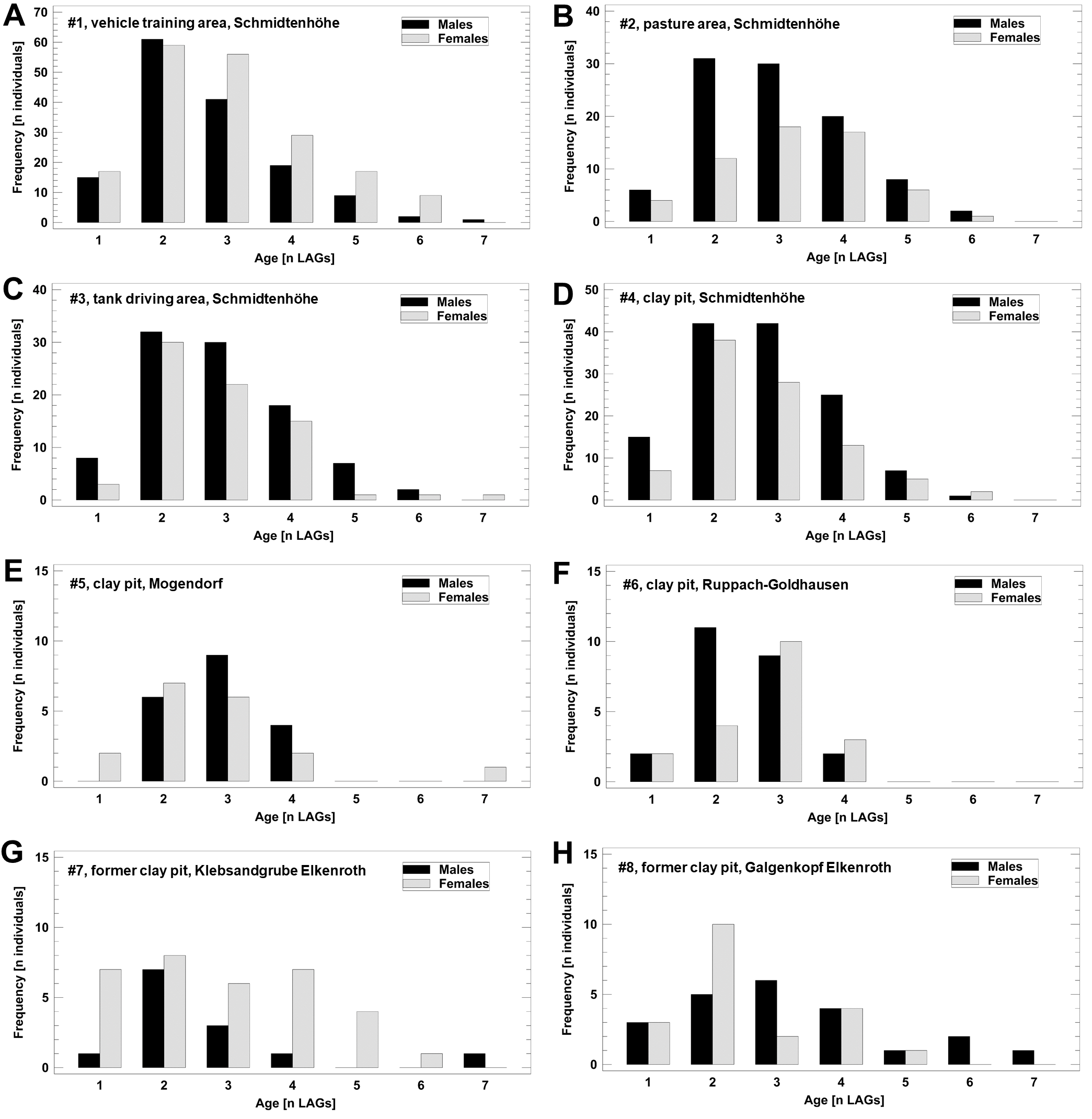

Supplement: Figure S1 — Data are pooled from all study periods at each locality (for details see Table 1). [file peerj-07-8233-s001.png]

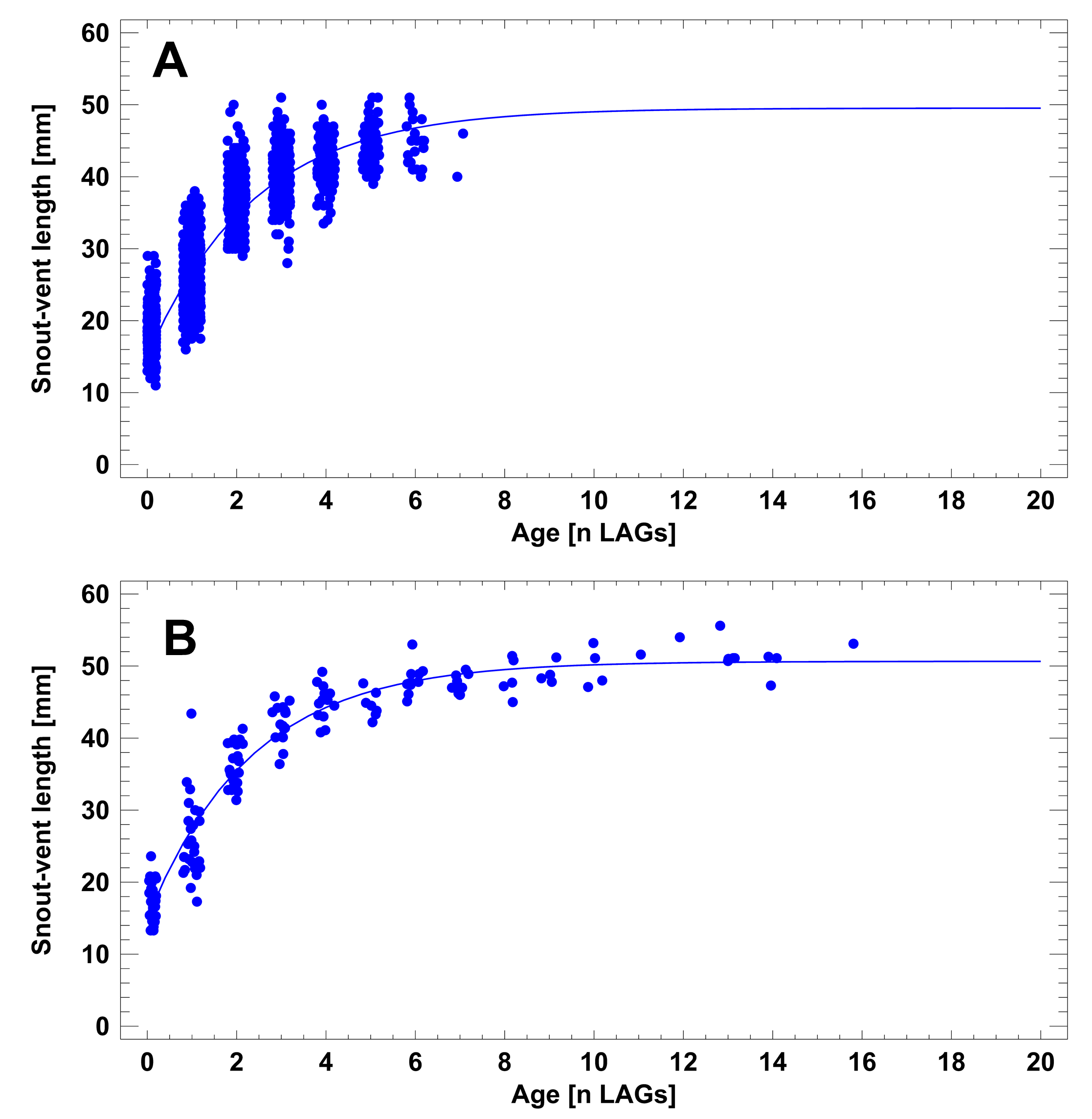

Supplement: Figure S2 — CRR versus (A) the lowest recorded air temperature during winter (December-February), and (B) the average air temperature during summer (June–August). [file peerj-07-8233-s002.png]

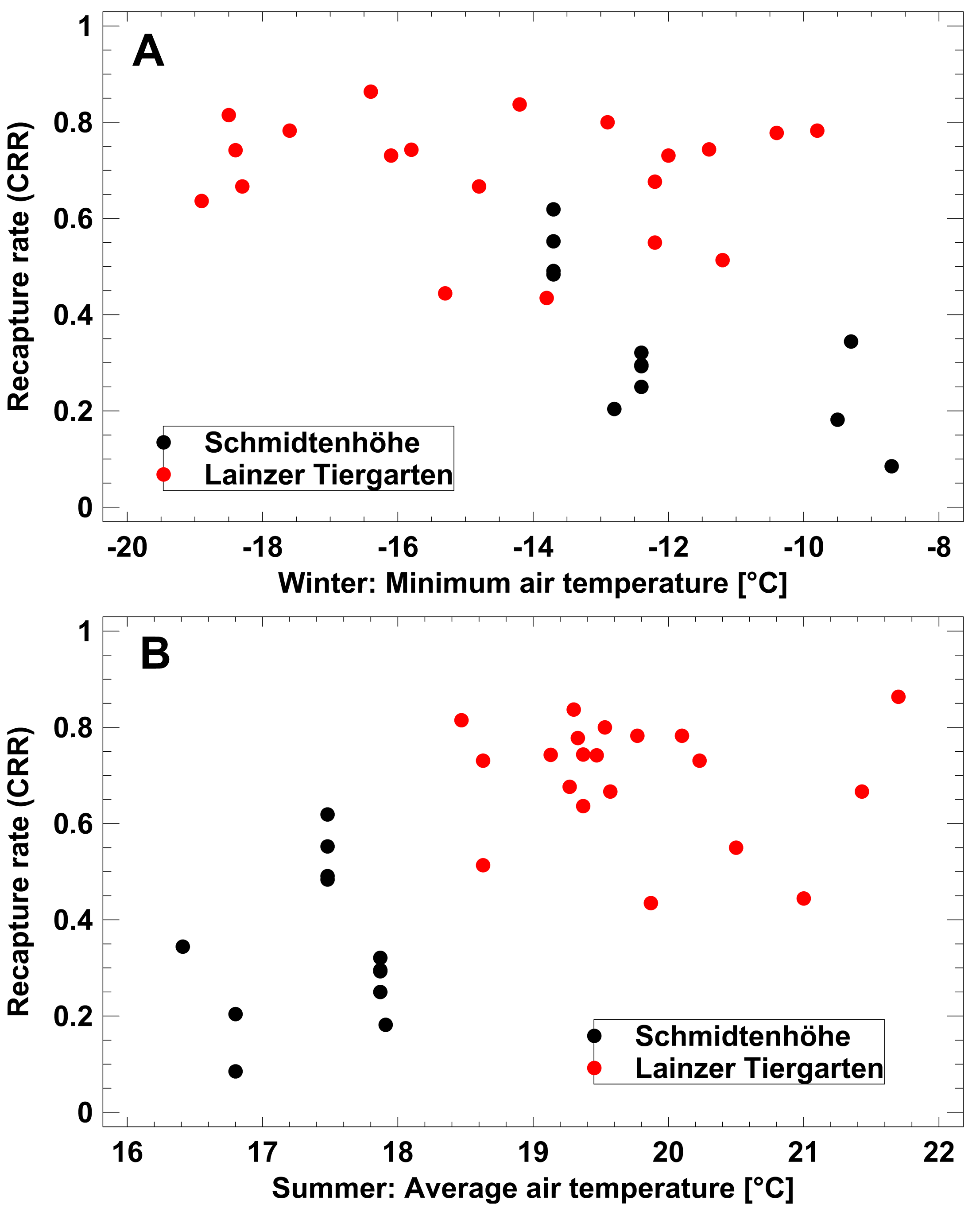

Supplement: Figure S3 — (A) Populations 1-8, Germany (model parameters: SVL_max= 48.6 mm [47.6-49.7 mm], k=0.442 [0.417-0.469]). Data are pooled from all study periods. (B) Population 9, Austria (model parameters: SVL_max= 50.6 mm [49.4-51.8 mm], k=0.433 [0.392-0.474]). [file peerj-07-8233-s003.png]
